# Case Report: Is there an age cutoff beyond which PFO closure should not be offered?

**DOI:** 10.3389/fcvm.2025.1622543

**Published:** 2025-08-29

**Authors:** Eustaquio Maria Onorato, Eleonora Melotti, Marco Doldi, Giovanni Monizzi, Angelo Mastrangelo, Vincenzo Mallia, Francesca Giacomazzi, Daniele Andreini, Antonio Luca Bartorelli

**Affiliations:** ^1^ University Cardiology Department, IRCCS Ospedale Galeazzi-Sant’Ambrogio, Milan, Italy; ^2^ Department of Biomedical and Clinical Sciences, University of Milan, Milan, Italy

**Keywords:** patent foramen ovale, right-to-left shunt, embolic stroke of undetermined source, aortic root dilation, ascending aorta elongation, arterial hypertension, case report

## Abstract

**Background:**

While randomized controlled trials have confirmed that patent foramen ovale (PFO) closure over medical therapy is considered the preferred treatment of young patients with embolic stroke of undetermined source (ESUS), the efficacy of percutaneous closure in elderly subjects with high-risk PFO remains unclear since no randomized trials are currently available.

**Case summary:**

A 65-year-old man with a past medical history of hyperuricemia, chronic gastroesophageal reflux disease, intestinal diverticulosis, and long-lasting arterial hypertension with enlargement of the aortic root was admitted for evaluation of the sudden onset of diplopia, postural instability, and subtotal loss of consciousness. Brain magnetic resonance imaging (MRI) confirmed an acute left thalamo-mesencephalic infarct. Continuous ECG monitoring ruled out atrial fibrillation (AFib). Two-dimensional (2D) transthoracic/transesophageal echocardiography (TTE/TEE) color Doppler showed a significant right-to-left shunt (RLS) via a tunnel-type PFO associated with hypermobile floppy septum primum (HSP) and prominent Eustachian valve (EV). Of note, a significant enlargement of the aortic root and ascending aorta resulted in a remarkable compression of the right atrial cavity and reduction of the interatrial septum (IAS) length, proportionally increasing its mobility and thus the amount of RLS. Following heart–brain team discussion, transcatheter PFO closure was recommended. After written informed consent, the patient underwent a successful percutaneous PFO closure with a self-expanding double-disk nitinol mesh PFO device (18/24 mm MemoPart) under local anesthesia and mild sedation, with fluoroscopic and rotational intracardiac echocardiography (rICE) guidance using a 9 F–9 MHz Ultra ICE catheter-based ultrasound probe. Two-month follow-up with 2D contrast TTE color Doppler and contrast-enhanced transcranial Doppler (c-TCD) showed correct device position, with no residual shunt.

**Discussion:**

Enlargement of the aortic root with increasing age may reorientate horizontally the IAS, allowing part of the flow to stream directly toward the PFO, decrease the size and length of IAS, and proportionally increase its mobility, thus uncovering latent or previously trivial RLSs in older hypertensive patients suffering from ESUS.

## Introduction

Randomized controlled trials (RCTs) (1–4) and several meta-analyses (5–7) have confirmed that transcatheter PFO closure over medical therapy alone is increasingly considered the preferred treatment for young patients who have had ESUS via paradoxical embolism with an overall improvement in quality of life. RCTs excluded patients over 60 years of age because older individuals often present with additional cardiac and vascular conditions—prevalent risk factors for stroke—while paradoxical embolism via patent foramen ovale is comparatively uncommon (8). At present time, the efficacy of percutaneous PFO closure in secondary prevention of stroke in patients older than 60 years is a matter of debate, and no randomized trials are currently available (9).

## Case presentation

**Figure 1:**
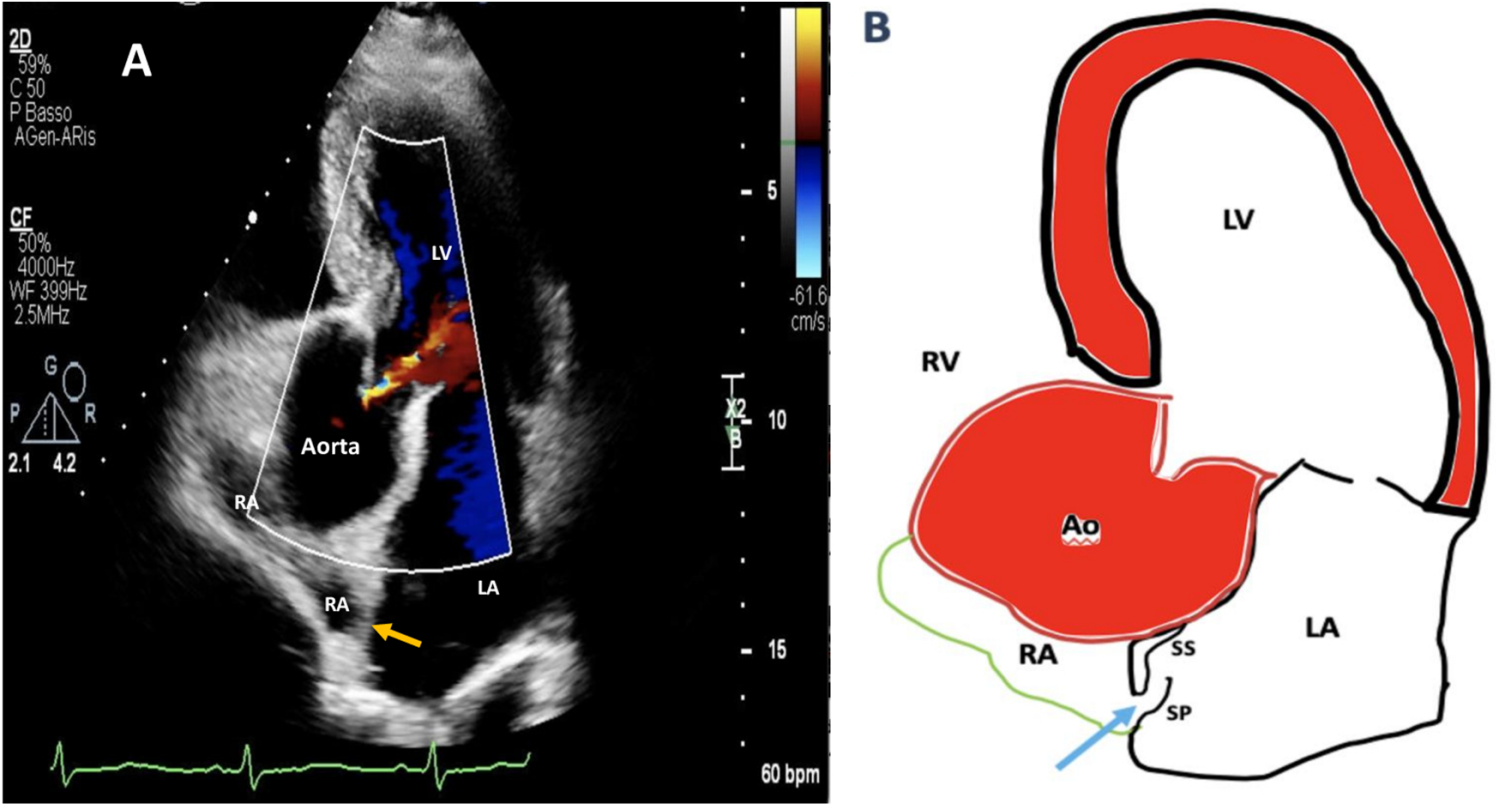
Two-dimensional (2D) transthoracic echocardiography (TTE) color Doppler apical five-chamber view showing aortic root dilation compressing remarkably the right atrial cavity and shortening the length of interatrial septum **(A)**. A simplified drawing showing compression of the RA by aortic root dilation: as the IAS basal diameter gets smaller, it becomes more mobile, the distance between septum primum and septum secundum increases, and this new configuration promotes right-to-left shunting throughout a manifest tunnel-like pathway (light blue arrow) **(B)**. RA, right atrium; LA, left atrium; LV, left ventricle; IAS, interatrial septum (orange arrow); SP, septum primum; SS, septum secundum.

We present a case of a 65-year-old man with a past medical history of hyperuricemia, chronic gastroesophageal reflux disease, intestinal diverticulosis, and concomitant long-lasting arterial hypertension associated with an enlargement of the aortic root. He was admitted mid-December 2024 to the Stroke Unit Department of a Tertiary Health Facility for evaluation of the sudden onset of diplopia, postural instability, and subtotal loss of consciousness. A comprehensive neurologic examination including close monitoring of early neurological deterioration was performed. Chest x-ray demonstrated aortic elongation, and a systolic murmur was detected at the left sternal border. Brain magnetic resonance imaging (MRI) confirmed an acute left thalamo-mesencephalic infarct. An accurate diagnostic workup for arrhythmias including 12-lead electrocardiogram (ECG) and 24–72 h dynamic Holter ECG monitoring ruled out paroxysmal atrial fibrillation (AFib). Notwithstanding, an implantable loop recorder (ILR) was inserted under the skin of the chest for longer cardiac monitoring. Two-dimensional (2D) transthoracic/transesophageal echocardiography (TTE/TEE) color Doppler showed left ventricular hypertrophy with normal systolic function, right-to-left shunt (RLS) via a tunnel-type PFO associated with HSP and Eustachian valve (EV). Enlargement of the aortic root (49 mm) and ascending aorta (44 mm), along with mild aortic incompetence, significantly reduced right atrial volume and interatrial septum (IAS) length, which increased interatrial septum (IAS) mobility (Figure 1, Supplementary Figures 1, 2) and changed heart angulation so that blood flow from the inferior vena cava (IVC) was more directly aimed at the PFO entry.

**Figure 2:**
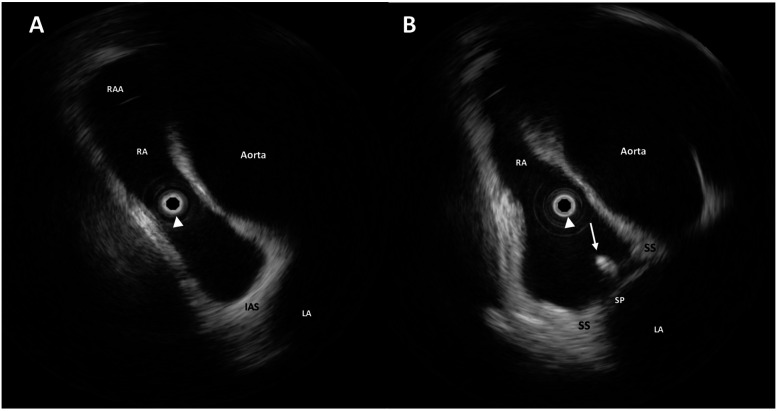
Intraprocedural rotational intracardiac echocardiography (rICE) using a 9 F–9 MHz Ultra ICE catheter-based ultrasound probe located at the center of the images (white arrowhead) in the aortic valve axial plane showing the enlargement of the aortic root and the ascending aorta shrinking the right atrium **(A)**. The guidewire (white arrow) crossing the interatrial septum through the PFO **(B)**. RAA, right atrial appendage; RA, right atrium; LA, left atrium; SS, septum secundum; SP, septum primum.

**Figure 3:**
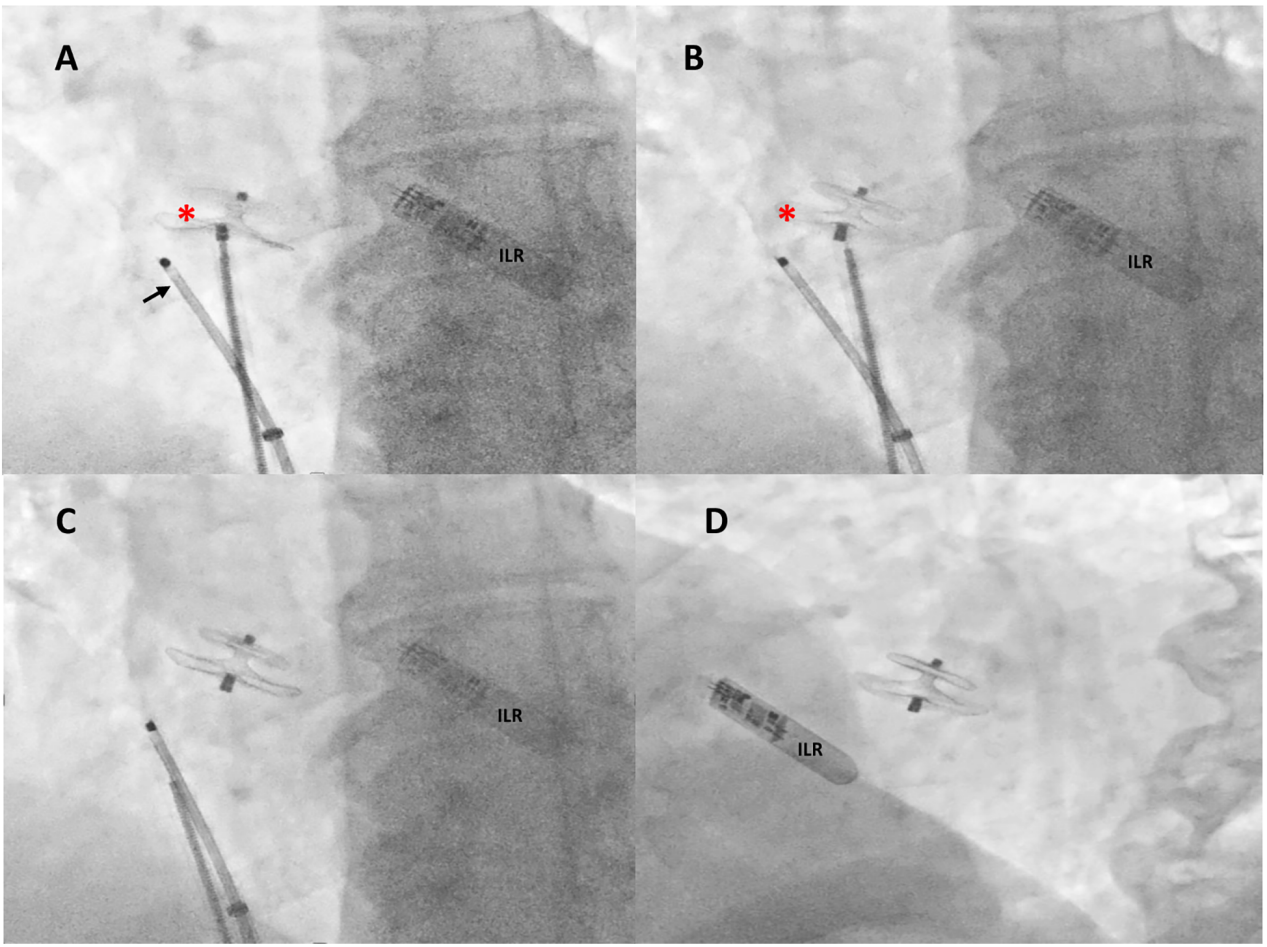
Intraprocedural fluoroscopic procedural steps. Under rotational intracardiac echocardiography (black arrow) and using a dedicated 9-F-long sheath, a 18/24 mm MemoPart PFO occluder (red asterisk) connected by a microscrew to a delivery cable was positioned across the PFO tunnel **(A)**, the device was then disconnected by turning the delivery cable counterclockwise (unscrewing) **(B)**, and the final correct position of the device was confirmed by fluoroscopy in anteroposterior **(C)** and left anterior oblique 30° view **(D)**. ILR, loop recorder.

**Figure 4:**
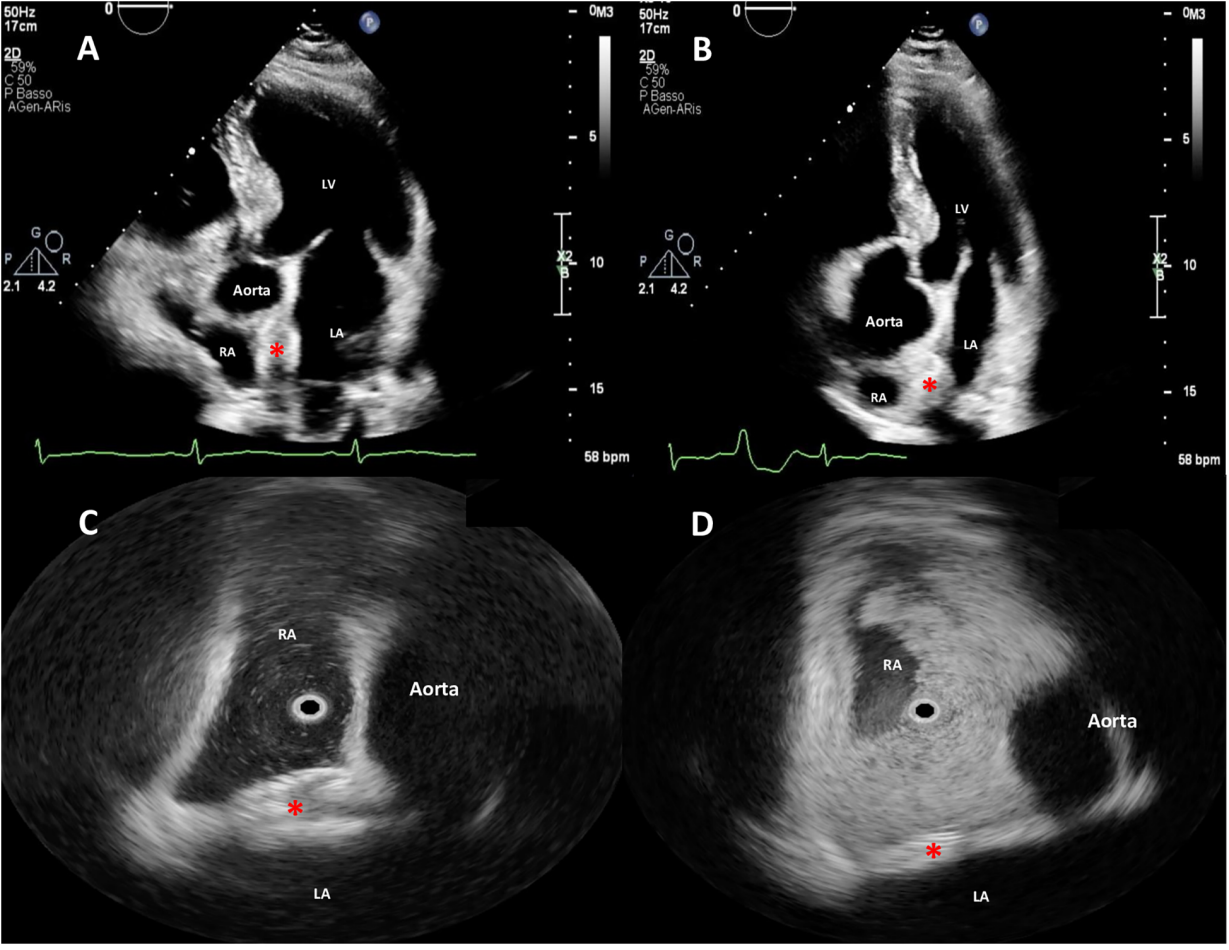
Two-dimensional (2D) transthoracic echocardiography (TTE) color Doppler **(A,B)** and rotational intracardiac echocardiography (rICE) **(C,D)** images demonstrating the final correct position of the PFO device (red asterisks), parallel to interatrial septum (IAS), not impinging the aortic wall, without residual shunt after agitated saline injection. RA, right atrium; LA, left atrium; LV, left ventricle.

A Doppler study of the lower limbs revealed no overt thrombosis. Ultrasound examination of extracranial blood-supplying arteries was normal, and carotid dissection was excluded. Thrombophilic disorders were ruled out. c-TCD showed a large RLS with >20 bubbles with a shower pattern in basal conditions, so Valsalva strain was not performed. Neurological symptoms progressively improved. Following heart–brain team discussion, transcatheter PFO closure was recommended, and the patient was transferred to our institution on 22 December 2024. After written informed consent, he underwent percutaneous PFO closure under local anesthesia and mild sedation, with fluoroscopic and rotational intracardiac echocardiography (rICE) guidance using a 9 F–9 MHz Ultra ICE catheter-based ultrasound probe (Boston Scientific Corporation, USA) (Figure 2) as previously described (10). Heparin (5,000 UI i.v.) was administered. A 9-F-long sheath was used to deliver an 18/24 mm MemoPart PFO occluder (Lepu Medical Technology, Beijing Co., Ltd.) connected by a microscrew to a delivery cable. From a femoral venous approach, the guiding sheath was passed through the PFO. Under fluoroscopic and rICE guidance, the device was advanced, and the small distal disk was released in the left atrium (LA) and pulled against the septum. The bigger right atrial disk was then deployed in the right atrium (RA) by pulling back the sheath, and finally the device was disconnected by turning the delivery cable counterclockwise (unscrewing) (Figure 3). Following device implantation, rICE demonstrated correct device placement with no residual shunt after injection of agitated saline solution (Figure 4). Moreover, ASA was completely stabilized and sandwiched between the two disks of the device. The patient was discharged home in good clinical condition on dual antiplatelet therapy with aspirin 100 mg/day and clopidogrel 75 mg/day for 6 months; endocarditis prophylaxis was also recommended. Persistent clinical improvement and no residual shunt at 2D TTE color Doppler were confirmed at 7-month follow-up.

## Discussion

Undoubtedly, enlargement of the aortic root in older hypertensive patients may decrease the size and length of the IAS, proportionally increase its mobility, allowing the unexpected appearance of RLS. Furthermore, it has been hypothesized that in PFO patients, an interaction exists between PFO size, RLS amount, and body position (standing or recumbent position). This interaction may be considered not only the result of dynamic changes in venous blood return to the right atrium but also possibly a consequence of the anatomic relationship between the interatrial septum and the aorta (11, 12).

Indeed, it has been largely demonstrated that enlargement of the aortic root may favor platypnea–orthodeoxia syndrome (POS) by creating and enhancing the amount of RLS (13, 14).

From a pathophysiological point of view, two mechanisms must go along to explain the establishment of RLS in our elderly patient. First is an anatomical primary defect such as interatrial communication, mainly represented by a PFO and less frequently by an ostium secundum atrial septal defect, with/without secondary anatomical variants such as ASA or EV, as in our case. Second is a functional factor which might cause interatrial septal deformation, redirection of shunt flow, stretching of an interatrial communication that occurs with a postural change as in platypnea–orthodeoxia syndrome (POS), and finally a decreased right ventricular compliance. Briefly, those functional factors can be vascular (aortic dilation or ascending aorta elongation), cardiac (constrictive pericarditis, right ventricular infarction), pulmonary (pulmonary arteriovenous malformation, pulmonary embolism, idiopathic pulmonary hypertension, severe chronic obstructive pulmonary disease, status/post pneumonectomy), abdominal (liver cirrhosis, diaphragm paralysis, abdominal surgery), and dorsal kyphosis (15).

Notably, aortic root dilation and elongation are very common in the adult population. The dilated ascending aorta as in our case was oriented horizontally, compressing the interatrial septum toward the right atrium to lead venous return from the inferior vena cava (IVC) to the interatrial communication (16–22). Eicher et al. suggested that a floppy atrial septum compressed by aortic root enlargement could act as a spinnaker in the venous blood flow; its billowing to the left would help to keep the foramen ovale wide open (23). Following ascending aorta enlargement, horizontal reorientation of the IAS plane that overlies the inlet of the inferior vena cava allows part of the flow to stream directly toward the interatrial communication (24). As the IAS basal diameter gets smaller, it becomes more mobile and more prone to shunting (12). These mechanistic factors are the most plausible explanation for our findings, and it is worth considering that patients with massive RLS tend to have larger aortic dimensions.

Although PFO prevalence appears to decrease, its size tended to increase with increasing age, from a mean of 3.4 mm in the first decade to 5.8 mm in the 10th decade of life (25). Age-related increase in risk of recurrent stroke associated with PFO is due to the increasing prevalence of venous thrombosis with age and consequent paradoxical embolization through the PFO, atherosclerotic risk factors, cardiac rhythm abnormalities specifically AFib, lower limb veins, degree comorbidities, increasing pulmonary pathologic changes, and right ventricular pressure potentially uncovering latent or previously nonsignificant RLSs and an increase in PFO size with age (26). However, it cannot necessarily be assumed that the proportion of strokes that are causally PFO-related is maintained at older ages, as the prevalence of other causes of stroke will tend to increase with age.

Kiblawi et al. reported that there was no significant difference in the rate of recurrent stroke/TIA after percutaneous PFO closure regardless of age (27). Spies et al. reported that the incidence of recurrent cryptogenic thromboembolic events after percutaneous PFO closure was not significantly different between patients above and below 55 years old after a median follow-up period of 18 months (28). However, these studies evaluated all causes of stroke recurrence, not only PFO-related cerebrovascular events. In addition, PFO morphology was unclear in these studies.

Takafuji et al., in their transcatheter closure of high-risk patent foramen ovale in the elderly, had an older sample (mean 75.2 ± 6.5 years old) and a longer follow-up period (mean 2.6 ± 1.8 years). Furthermore, only patients with high-risk PFO who underwent percutaneous closure were assessed for recurrence of PFO-related cerebrovascular events. Even though routine catheter-based closure in elderly patients with PFO-related paradoxical embolism should not be recommended because of the unclear outcomes associated with this therapy, high-risk PFO in older patients should be an indication to undergo percutaneous closure. Larger randomized trials are required to confirm these findings (29).

Ben-Assa et al. examined data on 741 patients (mean age 68 years) who underwent transcatheter PFO closure following a confirmed PFO-related stroke at a single center prior to 2018. Compared with younger patients, those older than 60 years old (*n* = 184) were more likely to have hypertension, smoking, and diabetes. Electrocardiographic monitoring (at least 2 weeks) prior to the procedure to rule out AFib was obtained in patients with CV risk factors and those over age 50. Device implantation was successful in 99% of the cohort, with similar rates of procedural success and complications between the younger and older patient groups. The rate of recurrent stroke or TIA was 4.3% at a median of 3.6 years in the older age group and 2.3% in the younger age group (*P* = 0.20). There was also a higher likelihood of developing AFib in older vs. younger patients (7.6% vs. 2.7%; *P* = 0.007). Nevertheless, in survival analyses recurrent ischemic neurologic events were not different by age (log-rank *P* = 0.31), and neither were the composite of ischemic neurologic events, reintervention, or neurologic death (log-rank *P* = 0.52). The authors concluded that monitoring for at least 2 weeks or longer with electrocardiographic monitoring such as an ILR is important in those over age 60 to rule out AFib, and there should also be a thorough assessment for carotid disease and other comorbidities that could be directly responsible for stroke (30).

The increased risk of new-onset AFib occurring usually within 45 days after percutaneous PFO closure was demonstrated in various studies (31). However, previous studies showed that approximately 75% of new-onset AFib episodes do not progress from paroxysmal to persistent and stroke caused by AFib related to PFO device closure is rare (32).

Recently, Mazzucco et al. (9) demonstrated that among the 153 patients found to have PFO (mean age 66.7 years), the risk of recurrent stroke was similar to that of a pooled estimate from a systematic review of 23 other studies (2.05 vs. 2.00 per 100 patient years) and the pooled ischemic stroke risk for patients 60 years and older was 3.27 per 100 patient-years. It 's worth noting that older patients with cryptogenic stroke/TIA and PFO may have a significantly higher risk of recurrent stroke than younger patients, with a nearly threefold increase at the age of 70 or above as compared with the mean age of patients enrolled in randomized closure trials. Nevertheless, this excess risk is specific as it is only seen for older patients with PFO when compared with patients of the same age without PFO. Finally, among patients with PFO and a history of stroke, older age is associated with a substantially higher risk of recurrent stroke. For decades, clinical research has excluded many high-risk patients over 60 from interventional studies.

## Conclusion

In summary, the interplay between anatomical predispositions—such as interatrial communication and associated variants—and functional factors such as vascular or cardiac alterations becomes increasingly relevant with age, especially in the context of aortic root enlargement and its hemodynamic consequences. Contemporary evidence underscores that while age does not markedly impact procedural success or major complication rates following percutaneous PFO closure, older adults may harbor greater comorbidities and display a heightened risk for atrial arrhythmias and recurrent cerebrovascular events. Thus, comprehensive evaluation—including extended cardiac monitoring and thorough assessment of alternative stroke etiologies—is essential in elderly patients with PFO-related events. Future large-scale, randomized studies focused on this population are warranted to clarify the optimal indications and management strategies in aging cohorts.

## Author's note

This article was the original work of the authors who have all seen and approved of the paper and authorship. The article has not been published elsewhere and is not under consideration in any other journals.

## Data Availability

The datasets presented in this study can be found in online repositories. The names of the repository/repositories and accession number(s) can be found in the article/Supplementary Material.

## Supplementary material

Supplementary Figure 1 Schematic representation of a tunnel-like PFO (functional tunnel length classification type I) with a normal interatrial septal length **(A)** and the distorted configuration with increased distance between septum primum and septum secundum **(B)** promoting an interatrial shunt in a case of shortened interatrial septal length by compression of the aortic root dilation. LA, left atrium; RA, right atrium; SP, septum primum; SS, septum secundum; ISL, interatrial septal length.

Supplementary Figure 2 Plate tectonics is a scientific theory that explains how major landforms are created as a result of Earth’s subterranean movements. Tectonic plates are composed of the oceanic lithosphere and the thicker continental lithosphere, each topped by its own kind of crust. Along convergent plate boundaries, the process of subduction carries the edge of one plate down under the other plate and into the mantle. The theory, which solidified in the 1960s, transformed the earth sciences by explaining many phenomena, including mountain-building events, volcanoes, and earthquakes.
