# Supplementary figures and images for: Case Report: Is there an age cutoff beyond which PFO closure should not be offered?
Source: Front Cardiovasc Med. 2025 Aug 29;12:1622543. doi: 10.3389/fcvm.2025.1622543 (PMC12426240; doi:10.3389/fcvm.2025.1622543)

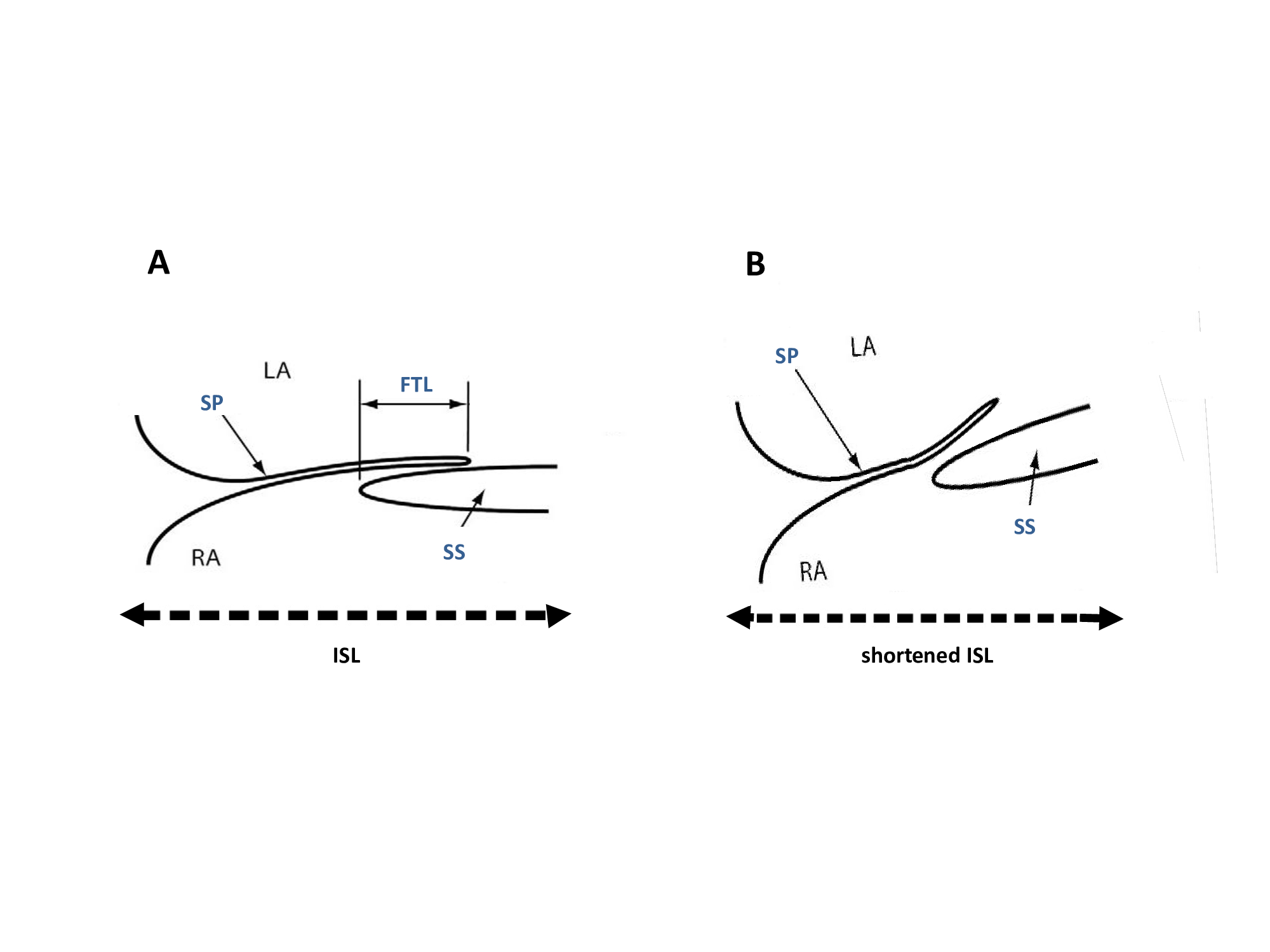

Supplement: Supplementary Figure 1 — Schematic representation of a tunnel-like PFO (functional tunnel length classification type I) with a normal interatrial septal length (A) and the distorted configuration with increased distance between septum primum and septum secundum (B) promoting an interatrial shunt in a case of shortened interatrial septal length by compression of the aortic root dilation. LA, left atrium; RA, right atrium; SP, septum primum; SS, septum secundum; ISL, interatrial septal length. [file Image1.jpeg]

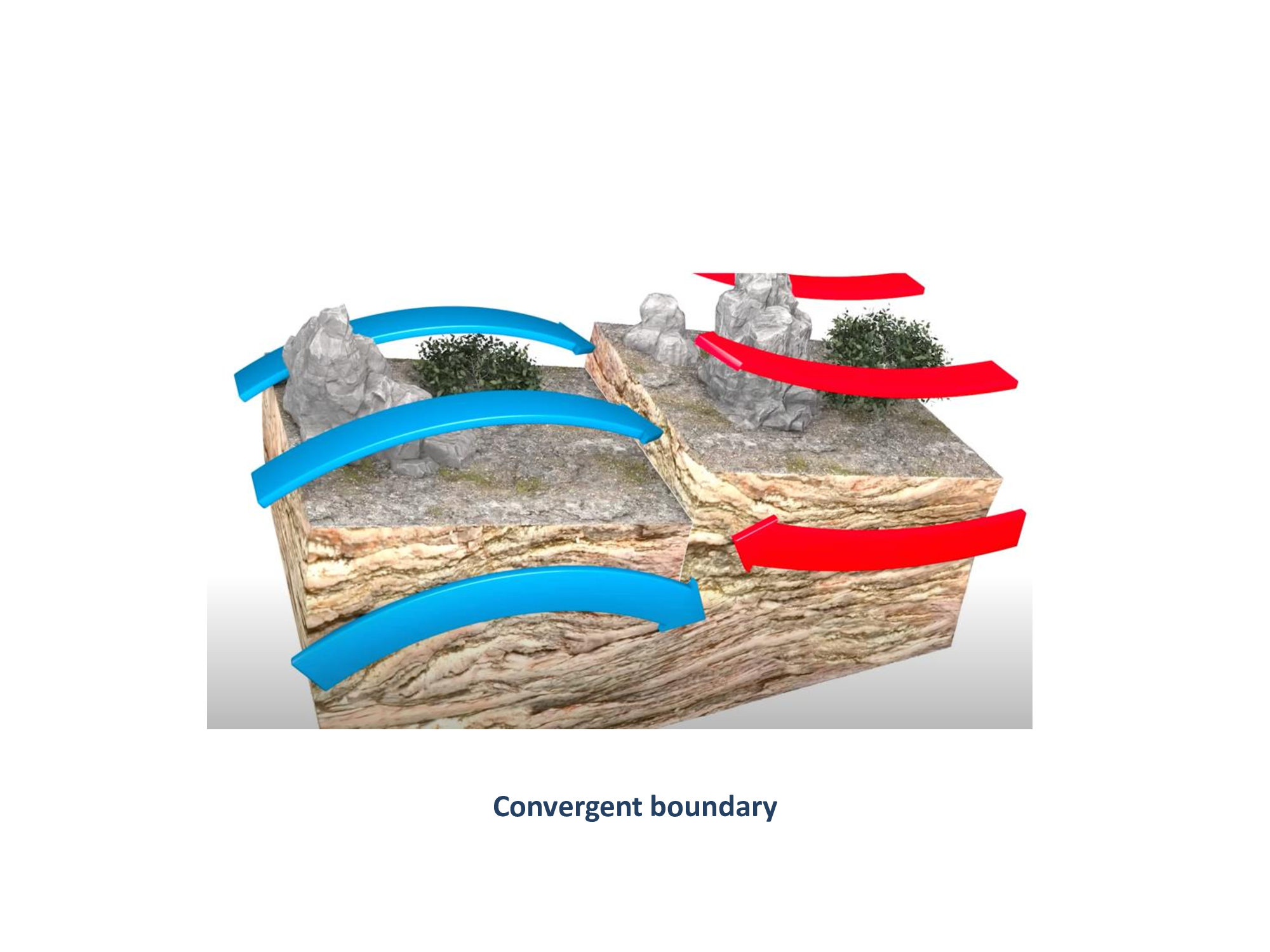

Supplement: Supplementary Figure 2 — Plate tectonics is a scientific theory that explains how major landforms are created as a result of Earth’s subterranean movements. Tectonic plates are composed of the oceanic lithosphere and the thicker continental lithosphere, each topped by its own kind of crust. Along convergent plate boundaries, the process of subduction carries the edge of one plate down under the other plate and into the mantle. The theory, which solidified in the 1960s, transformed the earth sciences by explaining many phenomena, including mountain-building events, volcanoes, and earthquakes. [file Image2.jpeg]
